# Green-synthesized lanthanum-doped CuO nanoparticles from *Embelia ribes* seed extract for dual electrochemical and photocatalytic applications

**DOI:** 10.1039/d6ra07883d

**Published:** 2026-11-10

**Authors:** Faiza Imtiaz, Usama Afzal, Qaisar Manzoor, Arfaa Sajid, Saira Zumar, Abid Ali, Imene Bayach, Amel Y. Ahmed, Warda Mansur

**Affiliations:** a Department of Chemistry, The University of Lahore 1-Km Defence Road Lahore Pakistan faiza.imtiaz@chem.uol.edu.pk abid.ali@chem.uol.edu.pk; b Department of Chemistry, College of Science, King Faisal University Al-Ahsa 31982 Saudi Arabia ibayach@kfu.edu.sa

## Abstract

*Embelia ribes* seed extract was used, for the first time, as a green chelating and reducing agent to synthesize lanthanum-doped copper oxide (La–CuO) nanoparticles. UV-vis screening of the La doping level (0–7%) identified 1% La as optimal, and Tauc plot analysis showed the band gap widened from 3.49 eV (CuO) to 3.72 eV (La–CuO). FTIR, SEM, XRD, and dynamic light scattering confirmed phytochemical surface capping, octahedral crystal morphology, a monoclinic CuO/cubic La_2_O_3_ structure (crystallite size 46 nm), and moderate colloidal stability (zeta potential −21.6 mV). La–CuO nanoparticles were evaluated for two applications: as an electrocatalyst, La–CuO-modified glassy carbon electrodes showed sensitive dopamine electro-oxidation by cyclic voltammetry and amperometry (*R*^2^ = 0.98 and 0.97, respectively), with roughly five-fold higher sensitivity than an unmodified electrode; as a photocatalyst, La–CuO degraded ∼49% of methylene blue within 24 h, establishing *E. ribes* as a sustainable bioresource for producing multifunctional, dual-application La–CuO nanomaterials.

## Abstract 2

*Embelia ribes* seed extract was used, for the first time, as a green chelating and reducing agent to synthesize lanthanum-doped copper oxide (La–CuO) nanoparticles. Image created with the assistance of Google Gemini.

## Graphical abstract

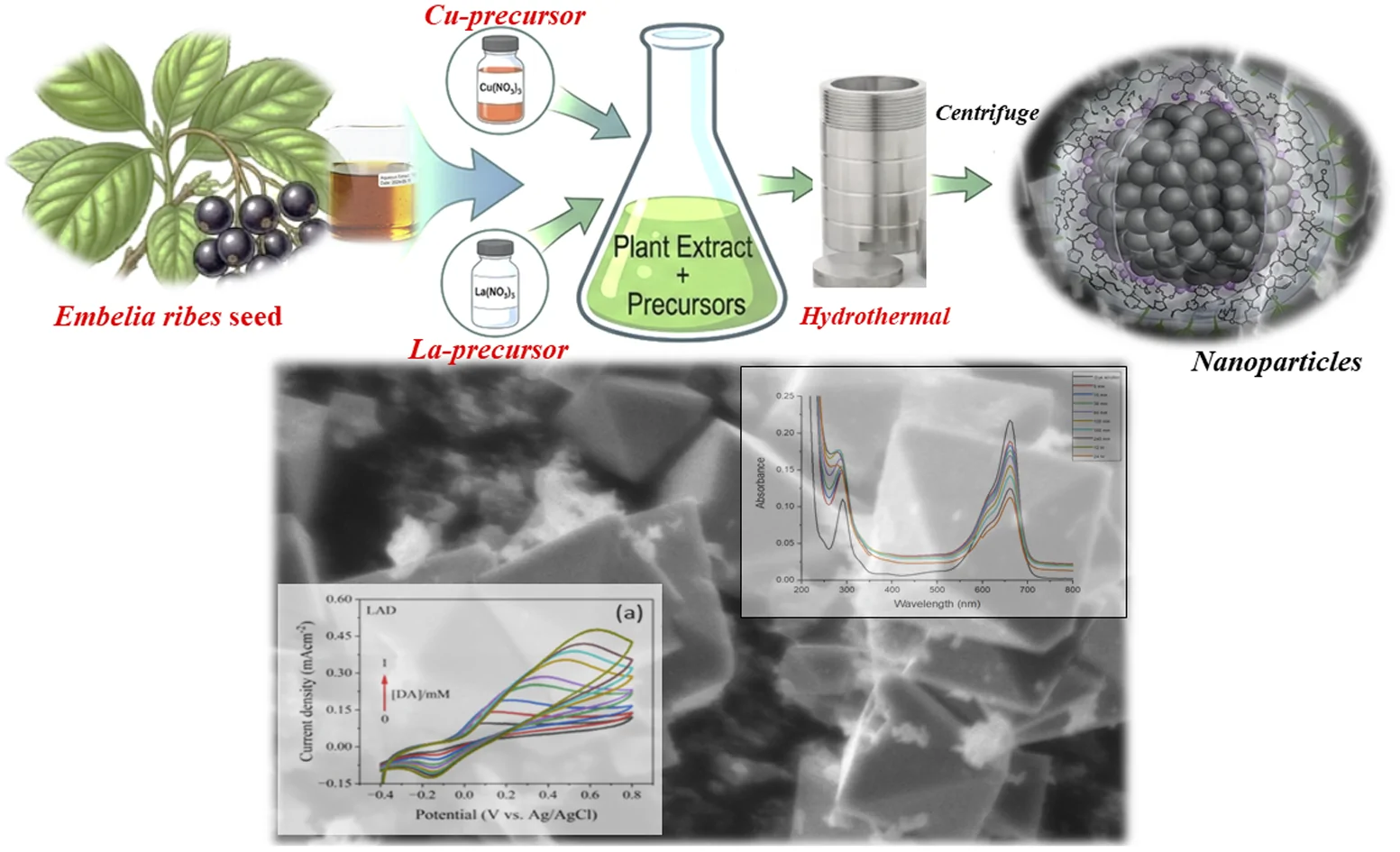

## 1. Introduction

Nanotechnology has emerged as a highly promising research frontier centered on the synthesis, characterization, and application of materials at the nanoscale.^1^ Nanoparticles-aggregates with dimensions typically between 1 and 100 nm-exhibit distinct physicochemical properties, including enhanced surface area, reactivity, and optical behavior, that differ markedly from their bulk counterparts, and these characteristics have driven the rapid expansion of nanoparticle-based technologies across cosmetics, food preservation, agriculture, targeted drug delivery, cancer diagnostics, catalysis, and chemical sensing. Among metal oxide nanoparticles, copper oxide (CuO) has attracted particular attention owing to its low cost, chemical stability, narrow band gap, and wide-ranging applications in catalysis, gas sensing, electrochemical sensing, and antimicrobial treatment. Doping CuO with rare-earth elements such as lanthanum (La) can further enhance its structural, optical, and electrocatalytic properties: La incorporation modifies surface defect density, electron mobility, and band gap, improving performance in photocatalytic, environmental, and electroanalytical applications.^2,3^

However, the synthesis of doped metal oxide nanoparticles conventionally relies on chemical reducing and capping agents that raise environmental and toxicity concerns, motivating growing interest in greener synthetic alternatives that exploit plant-derived phytochemicals as natural reducing and stabilizing agents.^4,5^ Such green routes have already been applied successfully to CuO nanoparticles using a range of botanical extracts, yielding materials suitable for applications spanning dye remediation, electrochemical sensing, and photocatalysis.^3,4^*Embelia ribes*, a medicinal plant of the Myrsinaceae family, has long been used in traditional Indian medicine for its anthelmintic, carminative, and stimulant properties. Its seeds are rich in bioactive compounds, including embelin (2.5–3.1%), quercitol (1.0%), fatty acids (5.3%), and trace volatile oils and tannins, and the growing interest in green synthesis has positioned *E. ribes* as a promising candidate for nanoparticle production, leveraging its phytochemicals as natural reducing and stabilizing agents; *Embelia*-derived nanoparticles have separately been explored for antioxidant, anti-inflammatory, and neuroprotective activity, bridging traditional herbal medicine with nanotechnology. More specifically, oxygen-rich phytochemicals such as embelin (2,5-dihydroxy-3-undecyl-1,4-benzoquinone), phenolics, and flavonoids are generally proposed to influence nanoparticle formation in three ways: (i) their hydroxyl and carbonyl groups can bind metal ions, forming complexes that control the rate of nucleation; (ii) phenolic groups can act as electron donors, being oxidized to quinone-type species, during the transformation of the metal ions; and (iii) the adsorbed phytochemicals can cap the growing particles, limiting aggregation and influencing particle size and morphology.^4,5^

Beyond environmental remediation, La-doped CuO nanoparticles are of growing interest as electrocatalytic materials for the electrochemical sensing of biologically important molecules. Dopamine (DA) is a key catecholamine neurotransmitter that regulates motor control, reward, and cognition; abnormal dopamine levels are implicated in Parkinson's disease and other neurological and psychiatric disorders, making its sensitive, rapid, and low-cost quantification clinically important.^6^ Electrochemical methods are attractive for dopamine detection owing to their simplicity, speed, and compatibility with miniaturized point-of-care devices, but unmodified carbon electrodes typically suffer from sluggish electron-transfer kinetics and overlapping oxidation potentials with co-existing electroactive species such as ascorbic acid and uric acid.^6,7^ Modifying electrodes with metal oxide and rare-earth-doped nanomaterials has been shown to lower the overpotential for dopamine oxidation, increase the density of active surface sites, and improve current response, and CuO- and La-doped CuO-based electrodes in particular have recently been reported as effective platforms for dopamine and related analytes.^8–10^ Similarly, the increasing discharge of synthetic dyes such as methylene blue from textile effluent poses a serious environmental threat, and conventional wastewater treatment methods often fail to fully remove these persistent pollutants. Nanoparticle-mediated photocatalysis, in which metal oxide and rare-earth-doped nanostructures generate reactive oxygen species under light irradiation to break down dye molecules, has emerged as an effective and comparatively low-cost remediation strategy, and green-synthesized, lanthanide-doped CuO nanoparticles in particular have been widely reported for efficient photocatalytic degradation of methylene blue and related dyes.^11–13^

Although La-doped CuO has previously been explored for photocatalytic degradation and bifunctional water-splitting electrocatalysis, the present study introduces a green, one-pot synthesis of La-doped CuO (La–CuO) nanoparticles using *E. ribes* seed extract as a natural chelating and reducing agent. The material was further optimized through systematic screening of La concentrations (0–7%), with 1% La identified as the optimum doping level for subsequent multifunctional applications. The resulting La–CuO nanoparticles were structurally and optically characterized by UV-vis spectroscopy, Tauc plot analysis, FTIR, SEM, XRD, and dynamic light scattering, and their dual functional performance was evaluated as (i) an electrocatalyst for the electrochemical sensing of dopamine at a modified glassy carbon electrode, using cyclic voltammetry, scan-rate-dependent kinetic analysis, and amperometry, and (ii) a photocatalyst for the degradation of methylene blue dye. Collectively, this work establishes *E. ribes* as a sustainable, underexploited bioresource for producing multifunctional La-doped CuO nanomaterials with practical relevance to electrochemical biosensing and environmental remediation.

## 2. Experimental section

### 2.1 Materials

*Embelia ribes* seeds were procured from a local herbal supplier and used as the plant source throughout this work. Copper(ii) sulfate (CuSO_4_), lanthanum nitrate La(NO_3_)_3_, sodium hydroxide (NaOH), Nafion (5 wt%) binder, methanol, ethanol, and methylene blue were of analytical grade and used as received without further purification. Distilled water was used throughout.

### 2.2 Preparation of seed extract from *Embelia ribes*

A total of 40 g of powdered *E. ribes* seeds was extracted using 500 mL of ethanol : water (70 : 30) v/v. Extraction was carried out by microwave-assisted sonication in 12 cycles of 10 s each (total 2 min) at 320 W. The extract was filtered through appropriate-grade filter paper to remove the solid residue, and the clear filtrate was collected. The resulting extract was stored at 4 °C prior to use.

### 2.3 Green synthesis of lanthanum-doped copper oxide nanoparticles

An aqueous solution of copper sulfate was prepared by dissolving 3.3 g of CuSO_4_ in 500 mL of distilled water. The *E. ribes* extract (10 mL) was introduced into this solution, followed by lanthanum nitrate (1 mol%) under vigorous stirring. The mixture was stirred continuously at room temperature (25 ± 2 °C) for 15–20 min without external heating. 1 M NaOH solution, prepared by dissolving 4 g of NaOH in 100 mL of distilled water, was added dropwise until precipitation occurred and the suspension became stable. The resulting suspension was transferred to a stainless-steel autoclave and subjected to hydrothermal treatment at 160 °C for 24 h to facilitate nanoparticle formation and crystallization under controlled conditions. The mixture was then centrifuged at 5000 rpm for 25 min, and the precipitate was collected and dried in a hot-air oven at 90 °C for 1 h to obtain the final black crystalline lanthanum-doped copper oxide (La–CuO) product. Nanoparticles with additional La loadings of 0 (undoped CuO), 3, 5, and 7 mol% were prepared by the same procedure to identify the optimum doping level (Section 3.1).

### 2.4 Characterization of La–CuO nanoparticles

#### 2.4.1 UV-visible spectral analysis

The optical properties of undoped and La-doped CuO nanoparticles were investigated over 200–800 nm using a double-beam UV-visible spectrophotometer (UV1900i) with a 1.0 cm path-length quartz cuvette. Samples were diluted with distilled water to optimize absorbance, and distilled water served as the blank for baseline correction. Tauc plots were generated from the UV-vis data for band gap estimation.

#### 2.4.2 Fourier transform infrared spectral analysis (FTIR)

Functional groups and chemical bonds were analyzed using a Bruker FTIR (model αT) spectrophotometer over 4000–500 cm^−1^.

#### 2.4.3 Scanning electron microscopy (SEM)

An FEI Nova 450 NanoSEM was used to examine nanoparticle topography and morphology.

#### 2.4.4 X-ray diffraction (XRD)

The crystal structure of La–CuO nanoparticles was characterized by XRD using a Bruker D2 Phaser diffractometer with Cu Kα radiation (*λ* = 1.54060 Å). Average crystallite size was calculated using the Scherrer equation (*D* = *Kλ*/*β*cos *θ*).

#### 2.4.5 Dynamic light scattering

The zeta potential of La–CuO nanoparticles was measured at 25 °C by dynamic light scattering (DLS) using a Malvern Zeta sizer Nano ZSP, with distilled water as dispersant.

### 2.5 Electrochemical sensing studies

#### 2.5.1 Fabrication of La–CuO@GCE

The La–CuO-modified electrode was prepared in three steps: (i) preparation of the catalyst ink, (ii) pretreatment of the glassy carbon electrode, and (iii) drop-casting and drying of the ink. La–CuO (20 mg) was dispersed in 20 µL distilled water and 20 µL methanol by sonication for 30 min, after which Nafion binder (5 wt%) was added and the mixture further sonicated to obtain a homogeneous slurry. Prior to modification, the glassy carbon electrode (GCE) was polished with alumina slurry and sequentially washed with dilute sulfuric acid, ethanol, and distilled water in an ultrasonic bath to remove surface impurities. A 5 µL aliquot of the La–CuO slurry was drop-cast onto the cleaned GCE and dried in an oven at 50 °C to obtain the La–CuO-modified electrode (La–CuO@GCE). The same pretreatment was applied to the bare GCE, which was then tested under identical conditions without the La–CuO layer. All current densities reported in this work are normalized to the geometric area of the GCE.

#### 2.5.2 Electrochemical measurements

Electrochemical measurements were performed using a conventional three-electrode system comprising La–CuO@GCE as the working electrode, Ag/AgCl as the reference electrode, and a platinum wire as the counter electrode, in 0.1 M phosphate buffer solution. Cyclic voltammetry (CV) was used to investigate the electro-oxidation behavior of dopamine (DA) over the potential window of −0.4 to 0.8 V *vs.* Ag/AgCl at increasing DA concentrations (0–1.0 mM). For comparison, an unmodified/bare GCE was evaluated under identical conditions as a reference system. Following optimization of the working potential from the CV data, amperometric (chronoamperometric) measurements were carried out at a fixed applied potential of 0.4 V *vs.* Ag/AgCl upon successive addition of dopamine under constant magnetic stirring, to evaluate the real-time sensing performance of La–CuO@GCE. The effect of scan rate (50–500 mV s^−1^) on the CV response was also examined to probe the reaction kinetics.

### 2.6 Photocatalytic dye degradation studies

#### 2.6.1 Degradation of methylene blue

Methylene blue (MB), a common cationic dye, was used as a model pollutant to evaluate photocatalytic degradation efficiency. A stock solution of MB (20 ppm) was prepared by dissolving 0.2 g of MB powder in 1000 mL of distilled water under constant stirring, and the solution was stored in an amber bottle to prevent photodegradation prior to use.

A predetermined quantity of La–CuO nanoparticles was added to the MB solution under continuous stirring to ensure uniform dispersion of the catalyst, and the suspension was allowed to react under ambient laboratory conditions. At regular intervals, aliquots were withdrawn, centrifuged to remove the nanoparticles, and analyzed by UV-visible spectroscopy (200–800 nm), monitoring the characteristic MB absorption peak at ∼664 nm. Percentage degradation of MB was calculated as:% *D* = [(*A*_0_ − *A*_*t*_)/*A*_0_] × 100where %*D* is the percent degradation, *A*_0_ is the initial absorbance, and *A*_*t*_ is the absorbance at time *t*. This method enabled systematic evaluation of the catalytic efficiency of the nanoparticles toward dye degradation and their potential for environmental remediation.

## 3. Results and discussion

### 3.1 UV visible spectroscopic analysis for optical properties

The formation of lanthanum-doped copper oxide (La–CuO) nanoparticles was first confirmed spectrophotometrically by recording UV-vis absorption spectra over the range of 200–800 nm. Both undoped CuO and 1% La–CuO nanoparticles exhibited a sharp absorption band at 220 nm (Fig. 1), attributable to charge-transfer transitions between O^2−^ (2p) and Cu^2+^ (3d) states, a signature typical of CuO nanostructures.^16^ Upon introduction of 1% La, an additional, intermediate absorption peak appeared at ∼320 nm that was absent in the undoped sample. The emergence of this feature indicates that La incorporation modifies the local electronic environment of CuO, introducing defect-related states and perturbing the band-to-band transitions of the host lattice.^14,15^

**Fig. 1:**
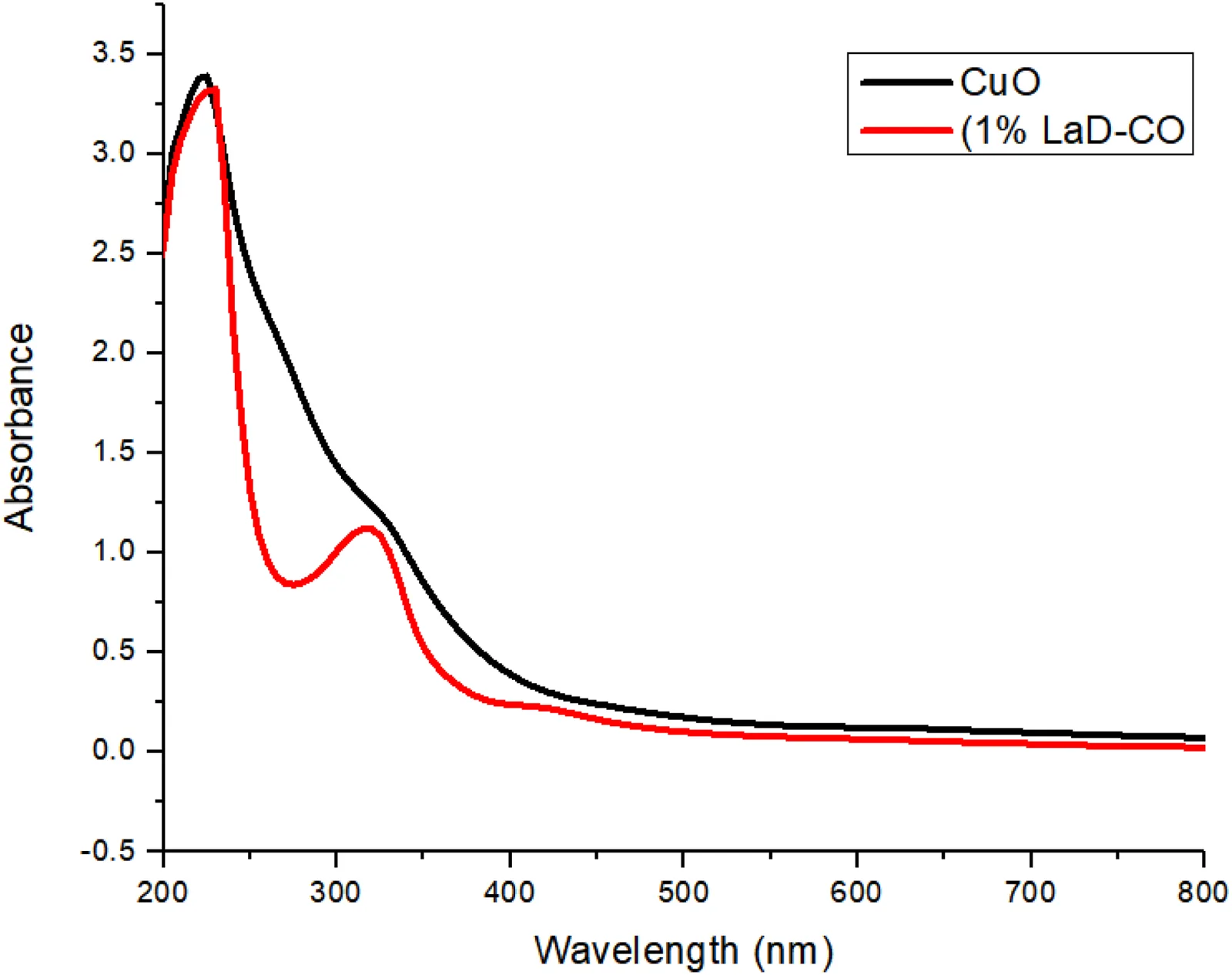
UV-vis absorption spectra of undoped CuO and 1% La–CuO nanoparticles.

Together, the retained 220 nm band and the newly resolved 320 nm feature suggest that lanthanum was successfully incorporated into the CuO framework without disrupting its characteristic optical signature, validating La–CuO formation at the 1% doping level. A UV-vis spectrum of a lanthanum-only reference was not recorded in the present study. However, La_2_O_3_ is a wide-band-gap oxide (*E*_g_ ≈ 5.5 eV) whose intrinsic absorption lies in the deep-UV region;^15^ the 320 nm feature is therefore unlikely to arise from an isolated La_2_O_3_ phase and is more reasonably associated with La-induced defect or interfacial states in CuO. La-only reference spectra will be included in future work.

To identify the optimum lanthanum content, CuO nanoparticles were additionally synthesized with increasing La loading (0, 1, 3, 5, and 7%) *via* the same green protocol, and their UV-vis spectra were compared (Fig. 2). The undoped sample (0% La) showed the highest primary absorbance, at ∼225 nm. Upon doping, the intensity of this primary band decreased relative to the undoped material, while a secondary absorption feature developed near 300–320 nm; this secondary peak was weakest for the 3% La sample, intermediate for 1% La, and most pronounced for the 5% and 7% La samples. Because higher La loadings did not further sharpen or blue-shift the characteristic CuO transitions, and higher dopant concentrations are more likely to promote defect scattering and partial particle agglomeration, 1% La was selected as the optimum doping level and used for all subsequent synthesis, characterization, and application studies reported in this work.

**Fig. 2:**
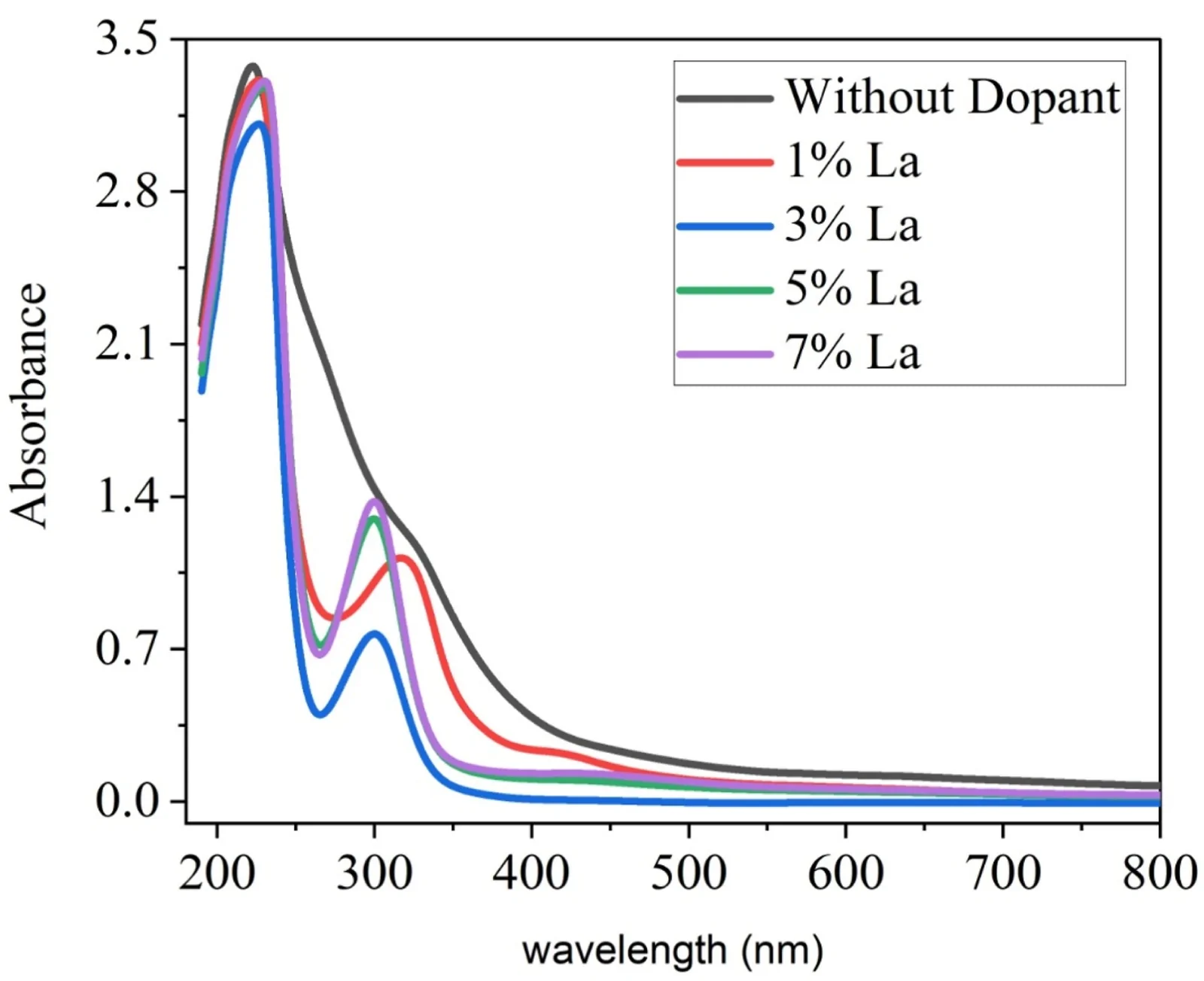
UV-vis absorption spectra of CuO nanoparticles synthesized with increasing La doping content (0, 1, 3, 5, and 7%), used to select the optimum La loading.

This trend is consistent with reports that excessive rare-earth doping in CuO can promote lattice strain, secondary-phase segregation, and light-scattering losses at grain boundaries, effects that suppress rather than enhance optical absorption. By contrast, a low doping level such as 1% La is more readily accommodated substitutionally within the CuO lattice, or as a well-dispersed secondary phase, preserving strong band-edge absorption while still introducing measurable defect-related electronic states. On this basis, the 1% La-doped sample (La–CuO) was carried forward for detailed structural, optical, and morphological characterization (Sections 3.2–3.6) and for evaluation of its electrochemical sensing and photocatalytic performance (Sections 3.7 and 3.8). This interpretation is consistent with the XRD results (Section 3.5), in which a separate cubic La_2_O_3_ phase is already detected at 1% La, indicating that the solubility of La^3+^ in the CuO lattice is limited. At higher loadings, a larger fraction of La is expected to segregate as La_2_O_3_, so the absorbance is not expected to change steadily with the nominal La content.

### 3.2 Optical band gap analysis

The optical band gap of the synthesized nanoparticles was determined from the UV-vis data using Tauc plots, based on the relation *E*_m_ = *hc*/*λ*, where *h* is Planck's constant, *c* is the speed of light, and *λ* is the wavelength of light. As shown in Fig. 3, the band gap increased from 3.49 eV for undoped CuO to 3.72 eV for 1% La–CuO.

**Fig. 3:**
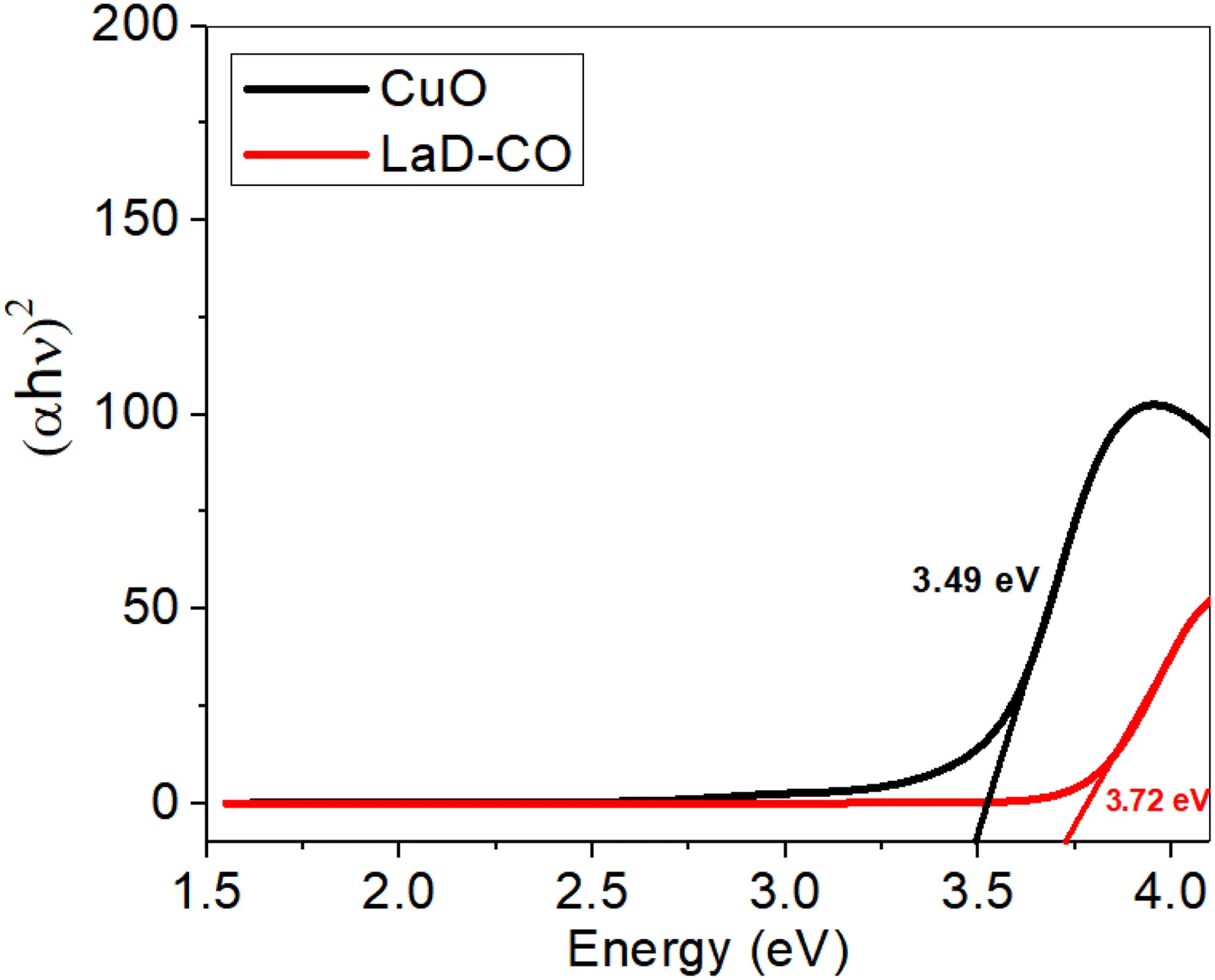
Tauc plots for band gap determination of undoped CuO and 1% La–CuO nanoparticles.

This widening of the band gap upon La doping can be attributed to several factors. Incorporation of lanthanum introduces additional electronic states into the CuO lattice, modifying the energy-level structure and altering the band structure of the host material. Because dopant incorporation can also influence nanoparticle size, a decrease in particle size would be expected to enhance quantum confinement effects, which likewise increases the band gap.^16^ The observed increase from 3.49 to 3.72 eV is therefore consistent with a combination of electronic-state modification and quantum-confinement effects arising from La incorporation.

### 3.3 Chemical composition analysis: FTIR spectroscopy

Fourier-transform infrared (FTIR) spectroscopy was used to identify functional groups present on the La–CuO nanoparticle surface and, by extension, the phytochemicals from *E. ribes* seed extract responsible for nanoparticle reduction and stabilization (Fig. 4). *E. ribes* seed extract contains embelin, a hydroxyl-benzoquinone reported to possess metal-binding/chelating properties, along with other phenolic and flavonoid constituents that may interact with metal ions. These phytoconstituents may therefore contribute to metal-ion complexation and nanoparticle stabilization during green synthesis, while their specific contribution to reduction remains hypothetical.

**Fig. 4:**
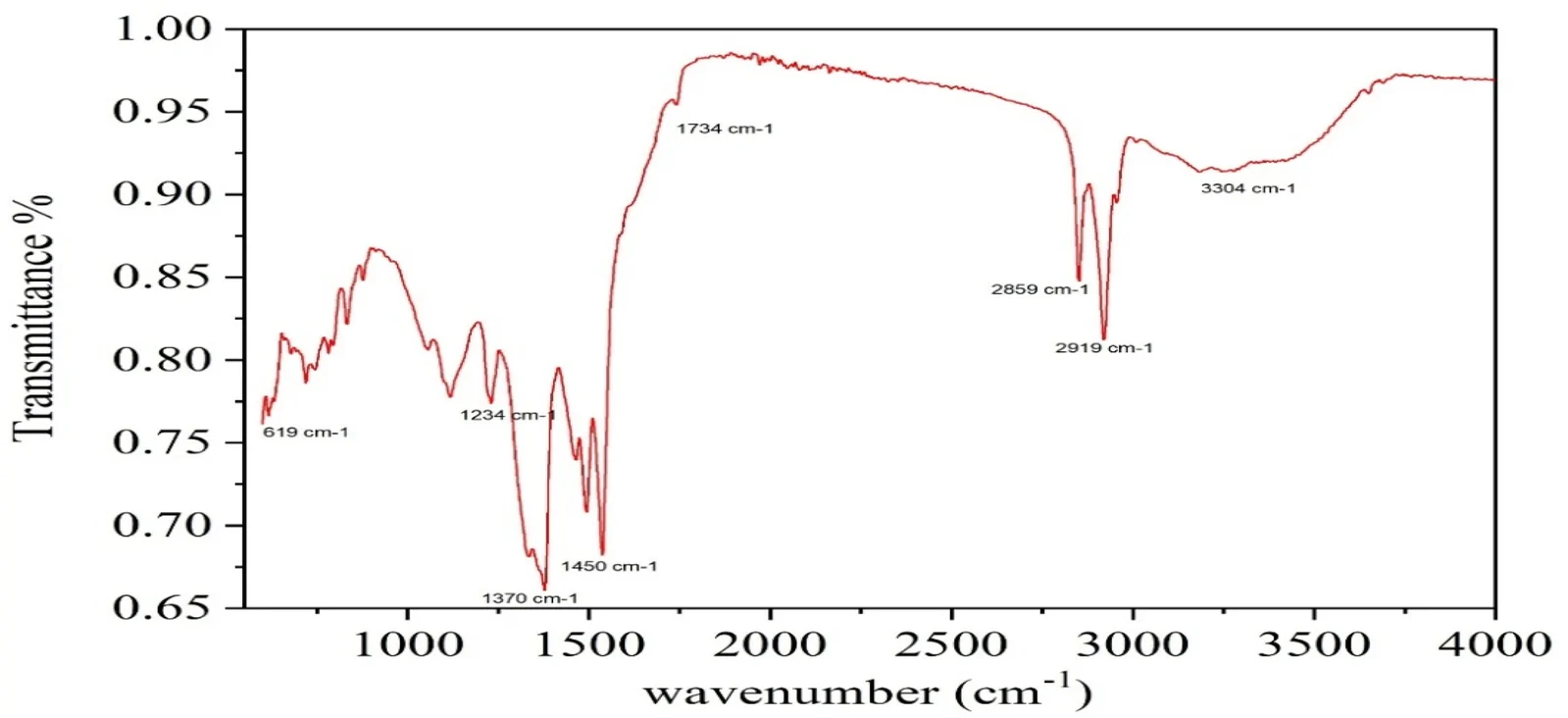
FTIR spectrum of La–CuO nanoparticles.

The band at 1234 cm^−1^ is attributable to C–N stretching vibrations, indicating the presence of amine- or amide-containing species, potentially alkaloids characteristic of *E. ribes*. The bands at 1370 and 1450 cm^−1^ correspond to C–H bending vibrations of methyl (CH_3_) and methylene (CH_2_) groups, suggesting the presence of aliphatic hydrocarbons such as fatty acids or terpenoids in the seed extract.^17^ The band at 1734 cm^−1^, assigned to C=O stretching of carbonyl groups (esters, aldehydes, ketones, or carboxylic acids), further supports the presence of carbonyl-containing phytoconstituents that may contribute to the biological activity of the extract.^18^

The bands at 2859 and 2919 cm^−1^ arise from C–H stretching vibrations of aliphatic CH_2_ and CH_3_ groups, consistent with lipid or fatty-acid components, while the broad band at 3304 cm^−1^ corresponds to O–H stretching of hydroxyl groups typical of alcohols, phenols, and carboxylic acids.^19^ The breadth of this band suggests hydrogen bonding characteristic of phenolic compounds, which are commonly associated with antioxidant activity and may contribute to the medicinal properties of *E. ribes* extract.^20^

In addition to these phytochemical-derived bands, a distinct absorption feature at 619 cm^−1^ was observed, assignable to Cu–O stretching vibrations characteristic of the CuO lattice. The presence of this metal–oxygen vibration, together with the organic functional-group bands described above, supports the formation of the CuO-based nanostructure and suggests the possible association of *E. ribes*-derived phytoconstituents with the nanoparticle surface. These phytoconstituents may hypothetically contribute to metal-ion interaction, reduction, and surface stabilization during the green synthesis, although their specific roles remain to be established. La incorporation was selected to modify the defect structure and charge-carrier behavior of CuO, with the aim of improving its functional properties. The *E. ribes* seed extract provides phytochemicals capable of complexing metal ions and facilitating their reduction and conversion into the corresponding oxide nanomaterial. The Cu–O band at 619 cm^−1^ agrees with literature values for CuO, while the La–O vibrations of La_2_O_3_ are reported mainly below ∼650 cm^−1^,^21^ where they overlap with the Cu–O band and lie partly outside the measured range (4000–500 cm^−1^). The presence of the La_2_O_3_ phase is therefore established from the XRD results (Section 3.5), and FTIR is used here primarily to identify the surface phytochemical functional groups.

### 3.4 Morphological analysis: SEM

Field-emission SEM imaging revealed that La–CuO nanoparticles formed well-defined, truncated-octahedral crystals with smooth facets and sharp edges, indicating a high degree of crystallinity (Fig. 5). This faceted morphology suggests that lanthanum ions act as a structure-directing agent during nucleation and growth: by selectively adsorbing onto high-energy crystallographic surfaces, La ions modify the surface-energy distribution and promote anisotropic growth, stabilizing the planes that favor octahedral geometries. This controlled growth pathway results in more uniform particles with fewer structural defects than typically observed for irregular morphologies.^22,23^

**Fig. 5:**
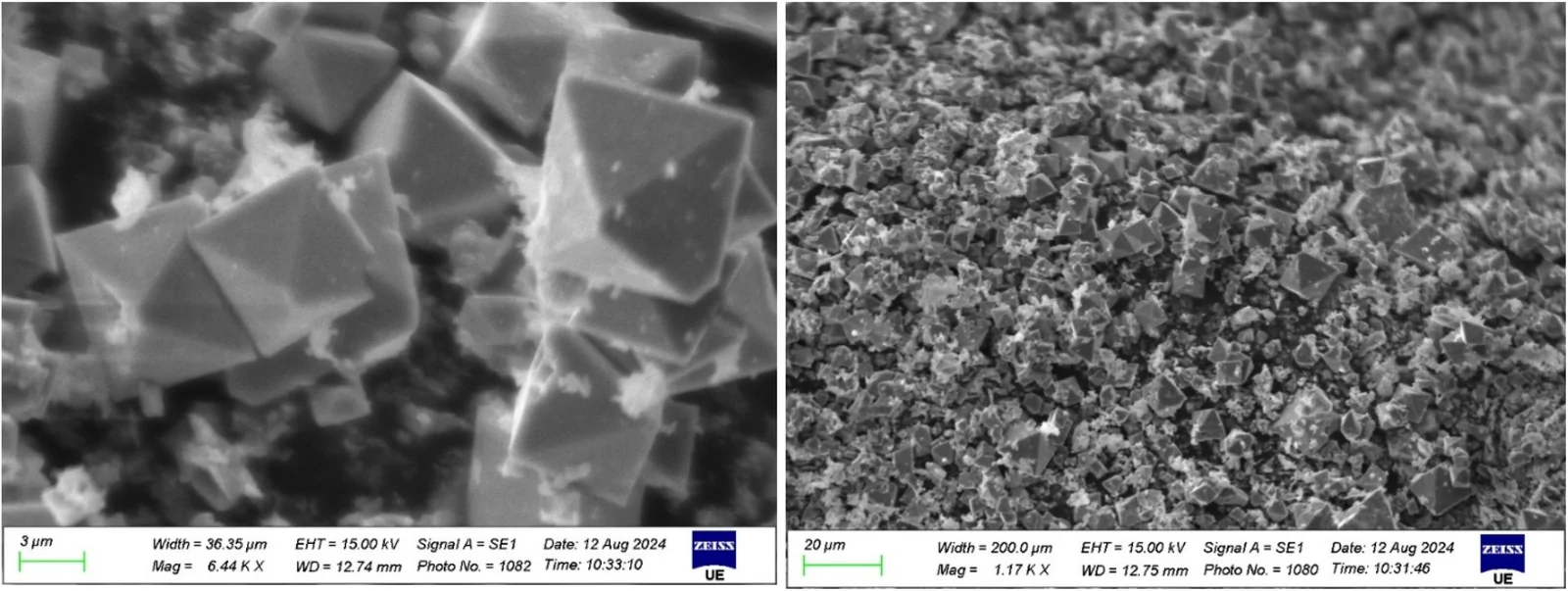
FE-SEM images of La–CuO nanoparticles under different resolution.

Beyond shaping particle morphology, lanthanum incorporation is expected to influence nucleation kinetics and surface properties, slowing nucleation and allowing gradual formation of larger, more uniformly faceted nanocrystals.^24^ Well-defined octahedral structures of this type are particularly advantageous for catalysis, sensing, and electronic-device applications, where surface area and crystal-facet exposure strongly influence performance,^25^ underscoring the relevance of the observed morphology to the electrochemical sensing and photocatalytic applications examined later in this work.^26,27^

### 3.5 Structural analysis: X-ray diffraction

The crystal structure of La–CuO nanoparticles was examined by XRD (Fig. 6). Diffraction peaks at 2*θ* values of 31.3°, 35.36°, 38.58°, 48.44°, 58.31°, 61.52°, 68.1°, and 75.04° were indexed to the (110), (−111), (111), (−202), (202), (−113), (−311), and (202) planes, respectively, of monoclinic CuO (JCPDS card no. 89-2531).^28^ The sharpness and moderate broadening of these reflections confirm the crystalline, nanoscale character of the material. Additional peaks at 20.6°, 26.89°, 37.2°, 40.60°, and 54.97° were indexed to cubic La_2_O_3_ (JCPDS card no. 22-0369).^29^

**Fig. 6:**
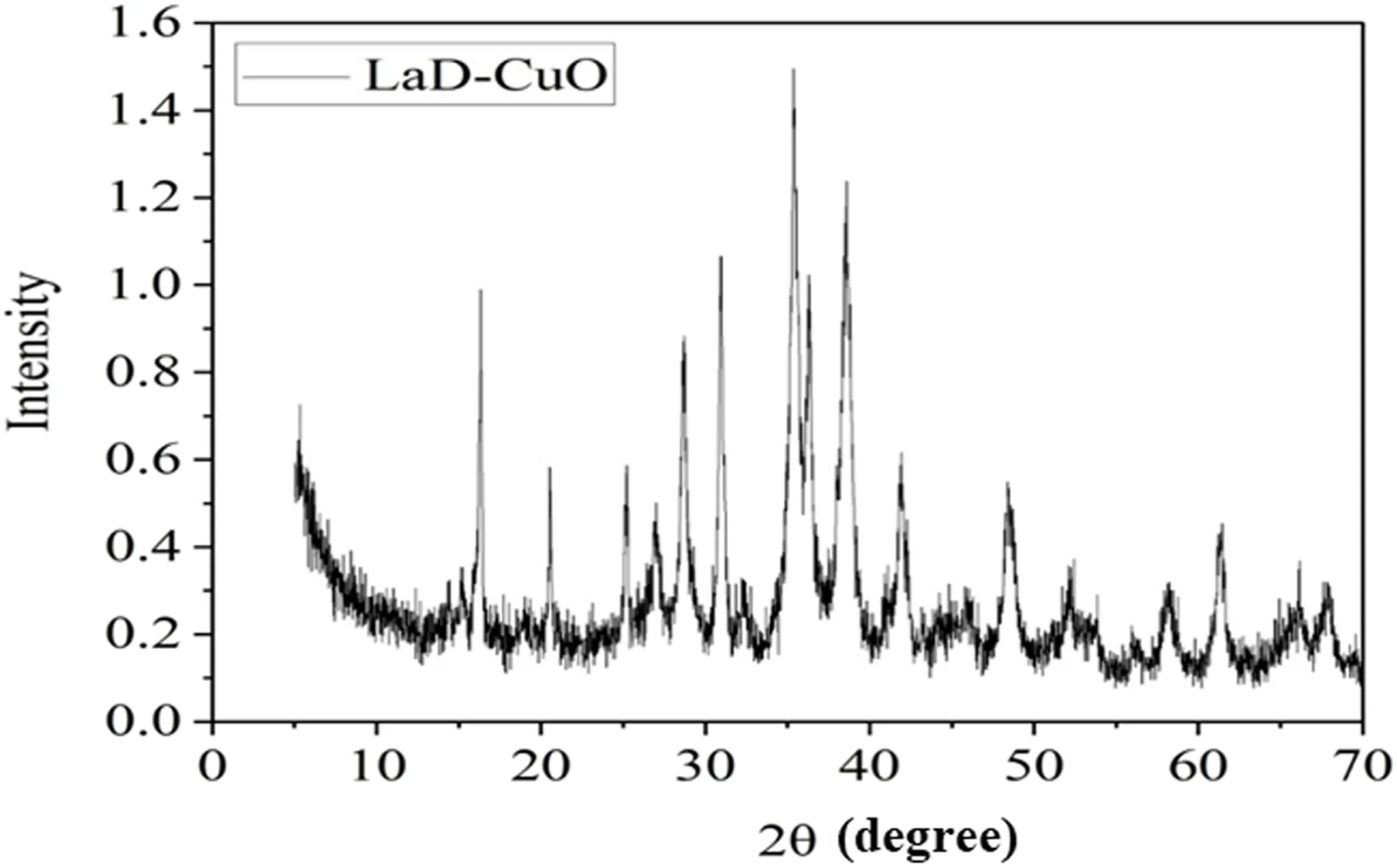
XRD pattern of La–CuO nanoparticles.

The presence of these La_2_O_3_-related reflections alongside the CuO peaks indicates that a substantial part of the lanthanum is present as a separate cubic La_2_O_3_ phase rather than being fully dissolved in the CuO lattice. Extensive substitution of Cu^2+^ (ionic radius ≈ 0.73 Å) by the much larger La^3+^ (≈1.03 Å) is unlikely because of this size mismatch, so the solubility of La in CuO is expected to be low. A small fraction of La may still occupies Cu sites or, more likely, reside at grain boundaries and CuO/La_2_O_3_ interfaces, where it can introduce the defect states suggested by the optical data (Sections 3.1–3.2). The material is therefore best described as La-modified CuO containing a La_2_O_3_ secondary phase. The average crystallite size, determined from the XRD pattern using the Scherrer equation, was 46 nm, confirming the nanoscale crystalline nature of the synthesized La–CuO material. The relatively small crystallite size may provide a high surface area and abundant surface-active sites, which can be beneficial for its photocatalytic and electrochemical applications.

### 3.6 Colloidal stability: zeta potential analysis

The zeta potential of the green-synthesized La–CuO nanoparticles was measured to evaluate their colloidal stability, since it reflects the degree of electrostatic repulsion between dispersed particles (Fig. 7). The nanoparticles exhibited a negative zeta potential of −21.6 mV.

**Fig. 7:**
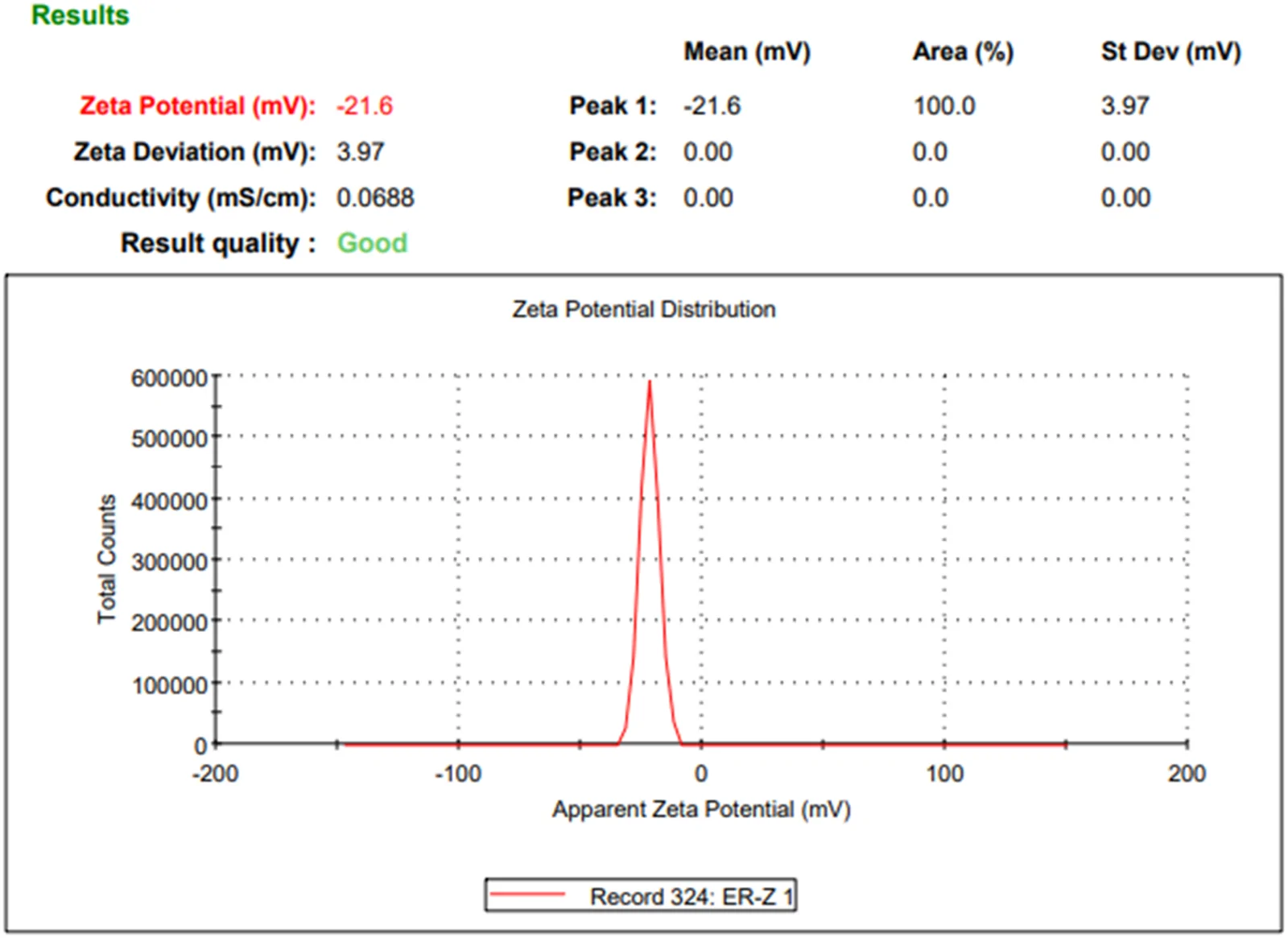
Zeta potential distribution of La–CuO nanoparticles.

Particles with zeta potential values outside the range of −30 to +30 mV are generally regarded as electrostatically stable; the measured value of −21.6 mV places the La–CuO nanoparticles close to this threshold, indicating moderate colloidal stability.^30^ This level of stability is considered adequate for the dispersion and surface accessibility required by both the electrochemical sensing and photocatalytic applications examined in this study.

### 3.7 Electrochemical sensing of dopamine

#### 3.7.1 Cyclic voltammetric response

The electrocatalytic activity of the La-doped CuO-modified glassy carbon electrode (La–CuO@GCE) toward dopamine (DA) oxidation was investigated by cyclic voltammetry (CV) and amperometry. As shown in Fig. 8a, La–CuO@GCE displayed a well-defined anodic response over the potential range of −0.4 to 0.8 V *vs.* Ag/AgCl, corresponding to the two-electron oxidation of dopamine to dopamine-*o*-quinone. Anodic peak currents increased systematically with rising DA concentration (0–1.0 mM), reflecting the highly active catalytic surface provided by La–CuO. A weak cathodic feature on the reverse scan indicated partial reduction of dopamine-*o*-quinone back to dopamine, consistent with quasi-reversible electrode kinetics governed in part by adsorption and intermediate accumulation at the electrode surface. The corresponding calibration plot (Fig. 8b) showed an excellent linear relationship between current density and DA concentration (slope = 0.41 mA cm^−2^ mM^−1^, *R*^2^ = 0.98).

**Fig. 8:**
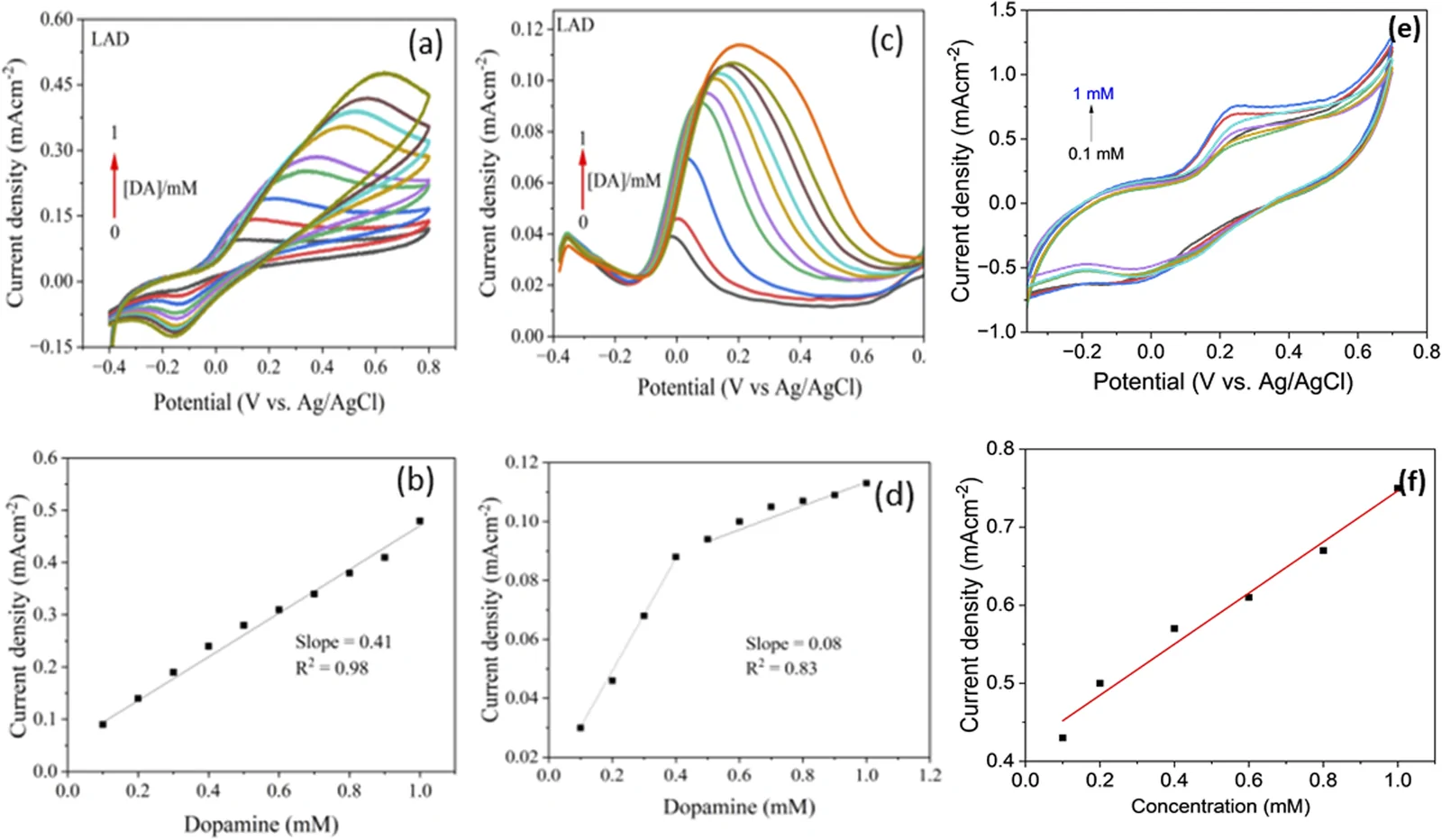
Cyclic voltammograms of (a) La–CuO@GCE (e) and bare GCE (c) square wave voltammetry of bare GCE in the presence of increasing dopamine concentrations (0–1.0 mM) with their corresponding (b, d, f) calibration plots.

For comparison, an unmodified/bare GCE was evaluated under identical conditions (Fig. 8c). This reference electrode produced substantially smaller current responses, with a shallower calibration slope (0.08 mA cm^−2^ mM^−1^) and weaker linearity (*R*^2^ = 0.83; Fig. 8d). The roughly five-fold increase in sensitivity for La–CuO@GCE relative to the bare electrode highlights the superior electrocatalytic activity imparted by La doping, which is expected to enhance surface active-site density, electrical conductivity, and defect-mediated charge transfer, thereby lowering the effective energy barrier for dopamine oxidation. Further CV analysis in (Fig. 8e and f) showed the lower performance at bare glassy carbon electrode in comparison with modified electrode.This behavior is consistent with recent reports describing enhanced dopamine-sensing performance at La- and other metal-doped CuO-based electrodes.^9,31,32^

#### 3.7.2 Effect of scan rate and reaction kinetics

The effect of scan rate on the electrochemical oxidation of dopamine at La–CuO@GCE was examined between 50 and 500 mV s^−1^ to probe the reaction kinetics. Both anodic and cathodic peak currents increased systematically with scan rate, while the overall shape of the voltammogram was preserved, indicating stable and efficient charge transport at the modified electrode. A gradual shift in peak potentials at higher scan rates pointed to decreased reversibility, consistent with quasi-reversible electron-transfer behavior.

The anodic peak current varied linearly with the square root of scan rate (*R*^2^ close to 0.97), in agreement with the Randles-Sevcik model,^33^ and indicating that dopamine oxidation at La–CuO@GCE is predominantly diffusion-controlled. Slight deviations from linearity at higher scan rates suggest an additional, adsorption-controlled contribution, consistent with interaction between dopamine molecules and defect-rich sites on the La–CuO surface. Overall, these results indicate a mixed kinetic mechanism in which diffusion dominates at low scan rates while surface adsorption becomes progressively more significant at higher scan rates, underscoring the role of La doping in promoting both rapid electron transfer and strong analyte adsorption.

#### 3.7.3 Amperometric sensing performance

To further evaluate the real-time sensing capability of La–CuO@GCE beyond the transient voltammetric response captured by CV, amperometric (chronoamperometric) measurements were performed upon successive addition of dopamine at a fixed applied potential of 0.4 V *vs.* Ag/AgCl under constant magnetic stirring (Fig. 9a). Each curve showed a sharp initial current spike immediately after addition of dopamine, followed by rapid decay to a stable plateau within approximately 10–15 s. This transient behavior reflects the near-instantaneous oxidation of dopamine at the electrode surface followed by establishment of a steady-state diffusion layer, after which the plateau current is governed by the rate of diffusive mass transport of dopamine to the electrode rather than by the electrode kinetics themselves. The clear separation between the plateau currents recorded for successive DA concentrations (0.6–1.0 mM) demonstrates that La–CuO@GCE resolves closely spaced concentration increments with minimal signal overlap, consistent with fast electron transfer and efficient renewal of the diffusion layer at the porous, faceted La–CuO surface.

**Fig. 9:**
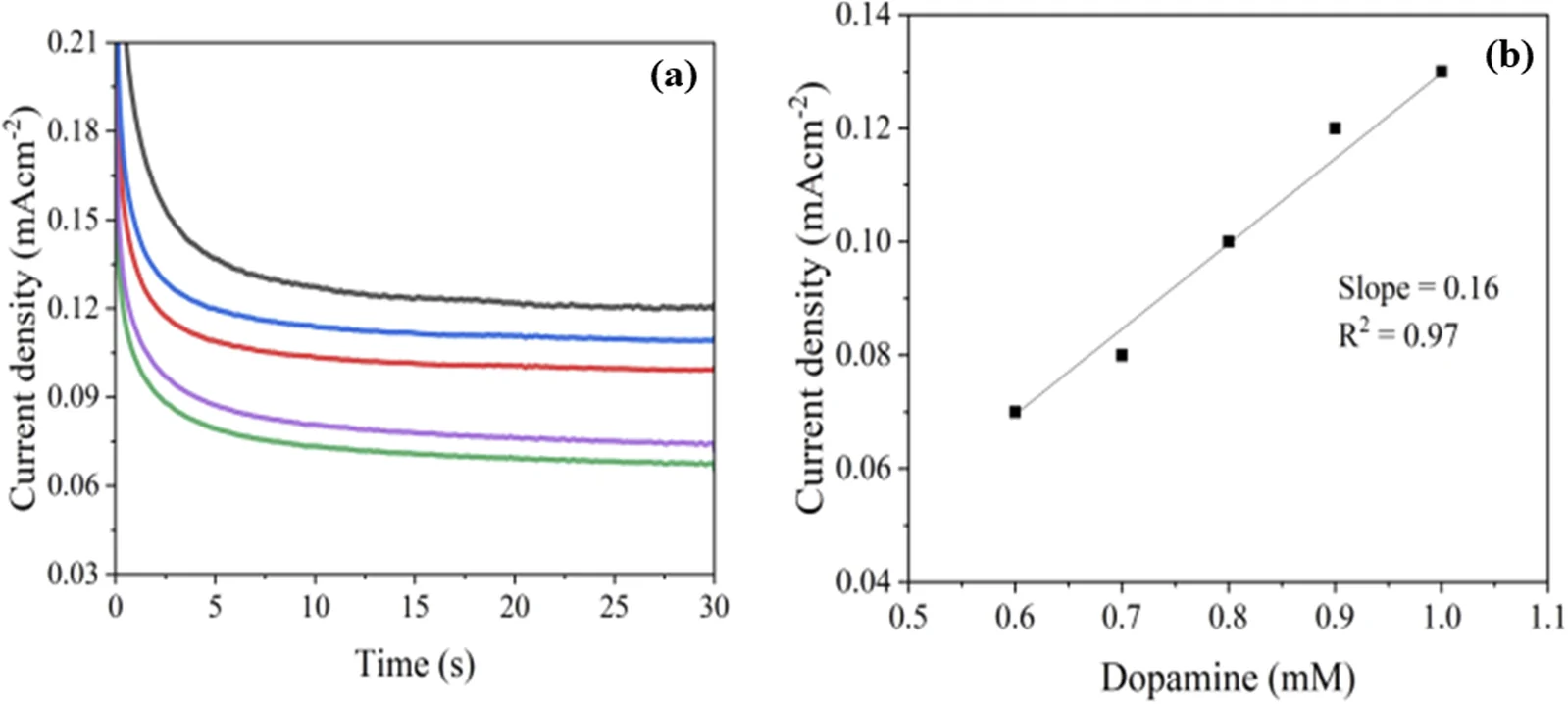
Amperometric response of La–CuO@GCE to successive additions of dopamine at an applied potential of 0.4 V *vs.* Ag/AgCl (a) and the corresponding calibration plot (b).

The corresponding calibration plot (Fig. 9b) exhibited a linear response with a slope of 0.16 mA cm^−2^ mM^−1^ and *R*^2^ = 0.97, confirming that the steady-state plateau current is diffusion-controlled over the concentration range studied. The amperometric sensitivity was lower than the value obtained by CV (0.41 mA cm^−2^ mM^−1^), which is expected given the different quantities the two techniques probe: CV measures a transient peak current that carries a mixed diffusion- and adsorption-controlled contribution (Section 3.7.2), whereas fixed-potential amperometry isolates the purely diffusion-limited steady-state response. This distinction is analogous to that reported for other CuO-based dopamine sensors, where amperometric and voltammetric sensitivities characteristically differ despite arising from the same electrocatalytic surface.^32,34^ Beyond sensitivity, the rapid stabilization time and low run-to-run variability of the current steps confirm the reproducibility and operational robustness of La–CuO@GCE, both of which are of direct practical importance for continuous, real-time sensing applications where a stable and quickly re-equilibrating baseline is essential. Taken together, the CV and amperometric data demonstrate that La–CuO@GCE combines well-defined redox behavior, high sensitivity, and good reproducibility across two complementary electroanalytical modes, supporting its potential as a practical dopamine-sensing platform.

Table 1 places these results alongside the bare-electrode control and a small selection of recently reported CuO-based dopamine sensors, to benchmark the performance of La–CuO@GCE against the wider literature. For direct comparability with the millimolar concentration range used throughout this study, all literature values are converted and reported in mM.

**Table 1:** Electroanalytical performance of La–CuO@GCE for dopamine sensing, benchmarked against the bare-electrode control (this work) and selected recently reported CuO-based electrodes

Electrode	Method	Linear range (mM)	Sensitivity (mA cm^−2^ mM^−1^)	LOD (mM)	Ref.
Bare GCE	CV	0.1–1.0	0.08	—	(This work)
La–CuO@GCE	CV	0.1–1.0	0.41	—	(This work)
La–CuO@GCE	Amperometry	0.6–1.0	0.16	—	(This work)
CuO:Mn/SPE	SWV	1 × 10^−4^ to 0.1	—	3.03 × 10^−5^	9
CuO@NDC/GCE	DPV	5 × 10^−3^ to 0.075	—	8.68 × 10^−4^	10
CuO/PANI (green)/GCE	SWV	0.026–0.095	—	8.22 × 10^−3^	32

Several features of the present work stand out against this benchmark. First, the roughly five-fold sensitivity gain of La–CuO@GCE over the bare GCE (Table 1, rows 1–2) is obtained on an internally matched pair of electrodes prepared and tested under identical conditions, giving a direct, self-consistent measure of the enhancement contributed by La doping-a comparison that is not always available in the literature, where a modified electrode is often benchmarked only against a differently configured bare surface. Second, La–CuO@GCE is validated by two independent electroanalytical modes, cyclic voltammetry and amperometry, both giving good linearity (*R*^2^ = 0.98 and 0.97, respectively), whereas each of the literature electrodes in Table 1 reports only a single technique. Third, and most distinctively, La–CuO nanoparticles are synthesized entirely from an aqueous plant extract without any synthetic surfactant, conducting polymer, or doped-carbon scaffold-in contrast to the CuO:Mn, CuO@NDC, and CuO/PANI platforms in Table 1 and the same La–CuO material is also shown in Section 3.8 to function as an effective photocatalyst, a dual utility not offered by any of the single-purpose sensors listed.

Sensitivity and limit of detection (LOD) are distinct figures of merit and are treated separately in Table 1. La–CuO@GCE was evaluated primarily for its sensitivity (current density per unit concentration) over a millimolar working range, and its LOD was not determined in the present study. This is because the calibration was performed only in the millimolar range (0.1–1.0 mM by CV and 0.6–1.0 mM by amperometry; Table 1), and lower dopamine concentrations and replicate blank signals were not recorded. An LOD derived from these data by the 3*σ*/slope method would require extrapolation far below the lowest concentration tested and would therefore not be reliable; by contrast, the CuO : Mn, CuO@NDC, and CuO/PANI electrodes were optimized for trace-level detection and report LOD values as low as 3.03 × 10^−5^ mM (30.3 nM), but their corresponding sensitivities were not reported in a directly comparable, area-normalized form. This trade-off reflects a difference in optimization goal rather than a shortfall of the present electrode: extending La–CuO@GCE toward a comparably low LOD is a clear direction for future work, achievable by narrowing the particle size, reducing catalyst loading, or coupling La–CuO with a conductive support, while retaining the green-synthesis and dual-functionality advantages demonstrated here.

### 3.8 Photocatalytic degradation of methylene blue

The photocatalytic performance of pristine CuO and La–CuO nanoparticles was evaluated for the degradation of methylene blue (MB) under ambient laboratory light. UV-vis absorption spectra recorded over the course of the reaction (Fig. 10) showed a progressive decrease in the characteristic MB absorption band at ∼664 nm with increasing contact time, confirming dye breakdown. After 24 h, La–CuO nanoparticles achieved 49% degradation of MB, demonstrating clear photocatalytic activity.

**Fig. 10:**
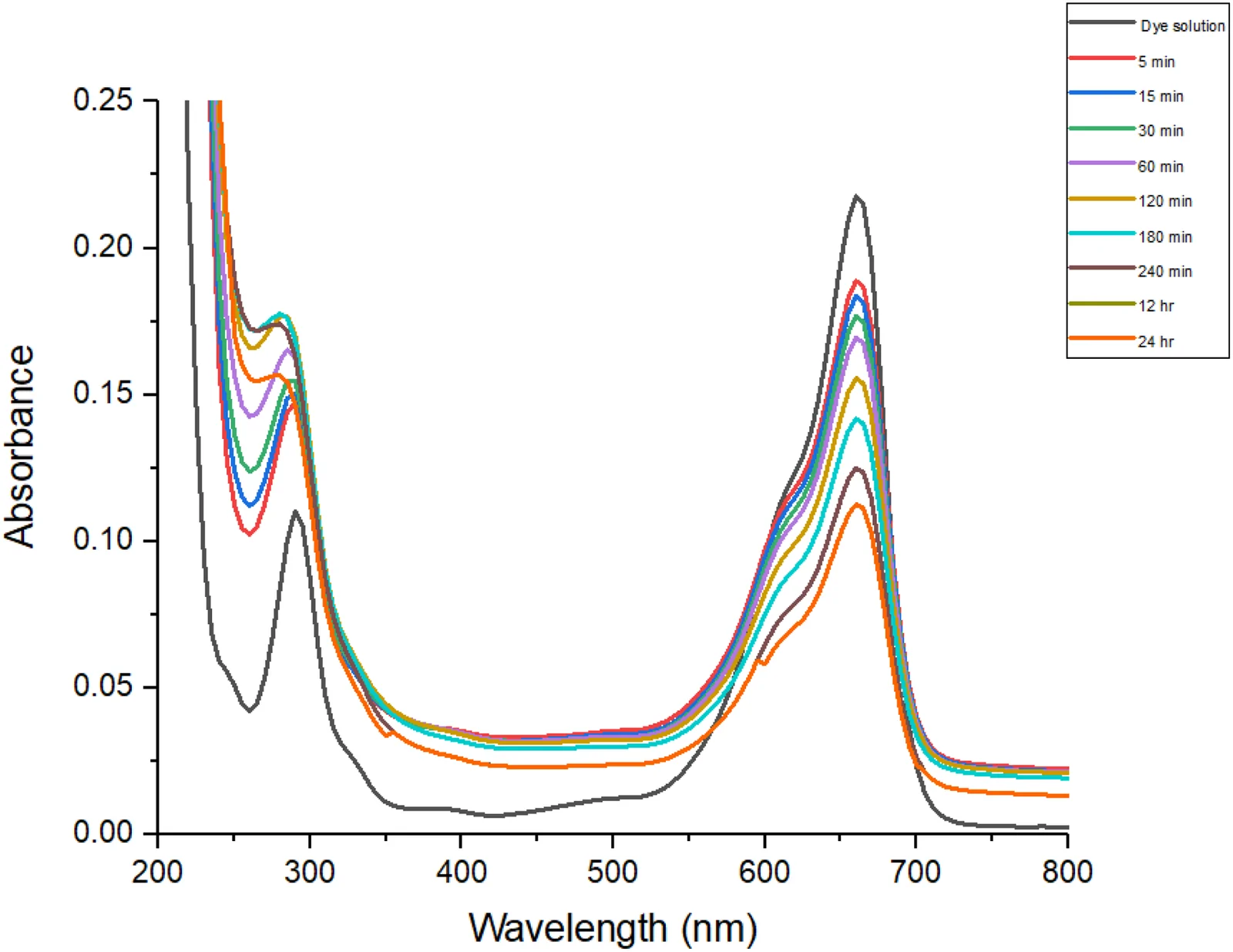
UV-vis absorption spectra showing the time-dependent degradation of methylene blue (MB) dye in the presence of La–CuO nanoparticles.

As noted in Section 3.2, La doping widened the optical band gap of CuO from 3.49 to 3.72 eV. While a wider band gap can in principle reduce visible-light absorption, the observed activity suggests that La incorporation instead improves photogenerated charge-carrier separation and suppresses electron–hole recombination, sustaining photocatalytic performance.^12,13^ La^3+^ substitution may also generate oxygen vacancies and surface defect sites that promote MB adsorption and reaction at the La–CuO surface. The proposed mechanism involves generation of reactive oxygen species (ROS) under light exposure: photogenerated electrons reduce dissolved oxygen to superoxide radicals (˙O_2_^−^), while photogenerated holes oxidize water or hydroxyl ions to hydroxyl radicals (˙OH); these ROS attack and degrade MB molecules, accounting for the observed decrease in absorbance with time.^12,13^

The photocatalytic activity of La–CuO was evaluated under ambient laboratory illumination, where 49% degradation of methylene blue (MB) was achieved after 24 h. This result indicates moderate photocatalytic activity rather than an optimized degradation performance. The relatively limited efficiency may be related to the wider band gap of La–CuO, which can restrict visible-light utilization, together with partial nanoparticle aggregation and electron–hole recombination. Since the experiments were conducted under uncontrolled ambient light conditions, parameters such as irradiation intensity, catalyst loading, reaction time, and light source were not systematically optimized. Therefore, the present result provides a preliminary demonstration of the photocatalytic potential of La–CuO, while further optimization under controlled irradiation conditions would be necessary to improve its degradation efficiency.^35^

The observed MB degradation can be tentatively attributed to the generation of photogenerated electron–hole pairs and subsequent formation of reactive oxygen species (ROS). The photogenerated electrons may interact with dissolved oxygen to generate superoxide radicals (˙O_2_^−^), whereas the holes may facilitate the formation of hydroxyl radicals (˙OH), which can participate in the degradation of MB.^36,37^ La incorporation may influence charge separation and the defect structure of CuO, thereby affecting the availability of reactive species. However, the individual contribution of the different ROS was not experimentally distinguished in the present study; therefore, the proposed degradation pathway should be considered tentative. Further mechanistic studies involving appropriate radical-scavenging experiments would be valuable for identifying the predominant reactive species and clarifying their contribution to MB degradation.^38,39^ Future studies should focus on optimizing the La doping level, catalyst loading, irradiation conditions, and reaction parameters. In addition, coupling La–CuO with narrow-band-gap semiconductors or suitable co-catalysts to construct heterojunction systems may enhance charge separation and further improve photocatalytic degradation efficiency.

## 4. Conclusion

In this study, a green and sustainable approach was developed for the synthesis of lanthanum-doped copper oxide nanoparticles using *Embelia ribes* seed extract as a natural source of chelating and reducing phytoconstituents. UV-vis screening of different La doping levels (0–7%) identified 1% La as the optimum composition. Spectroscopic, microscopic, and XRD analyses confirmed the formation of crystalline, well-defined faceted La–CuO nanostructures, with a zeta potential of −21.6 mV indicating moderate colloidal stability. La incorporation also increased the optical band gap from 3.49 eV for undoped CuO to 3.72 eV for 1% La–CuO. The synthesized nanoparticles exhibited multifunctional performance in both electrochemical sensing and photocatalysis. La–CuO-modified glassy carbon electrodes enabled sensitive, linear, and reproducible dopamine detection by cyclic voltammetry and amperometry, while scan-rate analysis suggested a combined diffusion- and adsorption-controlled oxidation process. In photocatalytic experiments, the nanoparticles achieved approximately 49% degradation of methylene blue after 24 h. Overall, the findings demonstrate the potential of *E. ribes*-mediated La–CuO as a low-cost and environmentally friendly multifunctional nanomaterial for dopamine sensing and dye remediation. Further studies should focus on selective multi-analyte detection, validation using real biological samples, and optimization of the photocatalytic process to improve degradation efficiency.

## Conflicts of interest

The authors declare no conflict of interests.

## Funding

This work was supported financially by the Deanship of Scientific Research at King Faisal University, under Ambient Researcher [Grant, KFU266099].

## Data Availability

The data that support the findings of this study are available from the corresponding author upon reasonable request.
